# Preoperative measurements on MRI in Chiari 1 patients fail to predict outcome after decompressive surgery

**DOI:** 10.1007/s00701-021-04842-y

**Published:** 2021-05-11

**Authors:** Miro-Pekka Jussila, Juho Nissilä, Minna Vakkuri, Päivi Olsén, Jaakko Niinimäki, Ville Leinonen, Willy Serlo, Niina Salokorpi, Maria Suo-Palosaari

**Affiliations:** 1 Department of Diagnostic Radiology, Oulu University Hospital, Oulu and Research Unit of Medical Imaging, Physics, and Technology, Oulu University Hospital and University of Oulu, Kajaanintie 50, OYS, P.O. Box 50, 90029 Oulu, Finland; 2 Department of Neurosurgery, Oulu University Hospital, Oulu and Research Unit of Clinical Neuroscience, Medical Research Center Oulu (MRC Oulu), Oulu University Hospital and University of Oulu, Oulu, Finland; 3 Department of Children and Adolescents, Oulu University Hospital, Oulu and PEDEGO Research Unit, University of Oulu and Medical Research Center Oulu (MRC Oulu), Oulu, Finland; 4 Department of Neurosurgery, Kuopio University Hospital, Kuopio and Institute of Clinical Medicine-Neurosurgery, University of Eastern Finland, Kuopio, Finland

**Keywords:** Chiari malformation, MRI, Preoperative, Postoperative, Decompressive

## Abstract

**Background:**

The purpose of our study was to research the parameters of magnetic resonance imaging (MRI) that would predict the outcome of surgery in patients with Chiari 1 malformation (CM1) and to evaluate changes in MRI parameters after surgery.

**Methods:**

Fifty-one patients (19 children, 13 adolescents, and 19 adults) operated on due to CM1 in Oulu University Hospital between 2004 and 2018 were evaluated. Seventeen parameters were measured from the preoperative MRI and 11 from the postoperative MRI. The correlations between the MRI parameters and the clinical variables before and after surgery were analyzed.

**Results:**

The majority (88.2%) of the patients had favorable surgical outcomes. Postoperatively, subjective symptoms improved in 88.6% of the patients and syringomyelia in 81.8%. The location of the cerebellar tonsils, when measured in relation to the C2 synchondrosis or the end plate, postoperatively moved cranially in 51.0% (*n* = 26), did not change in 27.4% (*n* = 14), and moved caudally in 21.6% (*n* = 11) of the patients. However, neither the location of the tonsils nor any other parameters measured from pre- or postoperative MRI correlated with the patients’ symptoms or surgical outcomes.

**Conclusions:**

No specific parameters on preoperative MRI evaluation were predictive of the outcome of surgery, emphasizing clinical examination in surgical decision-making. Furthermore, the postoperative MRI parameters did not correlate with the surgical outcomes. Thus, routine postoperative imaging is suggested only for patients with preoperatively diagnosed syringomyelia or worsening of symptoms.

## Introduction

Chiari 1 (CM1) is the mildest form of the posterior fossa malformation of the Chiari spectrum [24]. In CM1, the cerebellar tonsils are considered to be located at least 5 mm below the foramen magnum [4, 13, 31, 36]. However, in infants, the diagnostic threshold is considered to be at least 7 mm below the foramen magnum [6]. CM1 is a usual incidental finding in magnetic resonance imaging (MRI), and it is found in 0.24–0.9% of the adult population [28, 38]. The prevalence of CM1 in children is reported to be in general 1.0–3.6% [1, 33] and 0.6–0.8% in purely asymptomatic children [10, 17]. The subjective symptoms of CM1 vary from headache to weakness or paralysis of the limbs [4, 22]. No reliable parameters have been found in MRI studies that correlate with clinical symptoms [4].

Syringomyelia and scoliosis are considered to be associated with CM1 [4, 36]. The prevalence of syringomyelia in patients with CM1 varies between 20 and 70% [18, 33, 36]. The customary treatment for CM1 is surgical decompression [41]. However, not all patients will benefit from surgery [2, 25–27]. A wide variety of imaging parameters have been described for evaluating the severity of CM1 to aid in surgical decision-making and assessing surgical outcome [3, 4, 16, 25, 27, 31, 36]. However, reliable radiological parameters have not yet been found to evaluate which cases will benefit from surgery [4]. In some studies, the preoperative presence of syringomyelia was associated with better surgical outcomes, while in other studies, no significant difference was observed between the patients with or without syringomyelia [4, 18]. Our aim was to determine which MRI findings could predict a favorable surgical outcome.

## Methods and materials

### Patient population

This retrospective study included all the patients operated on due to CM1 in Oulu University Hospital between 2004 and 2018 who had both preoperative and postoperative MRI images available. Patients operated on due to CM2 or stenosis of the foramen magnum without cerebellar tonsillar descent and patients with secondary CM1 due to craniosynostoses were excluded. During these 15 years, 59 patients were operated on due to CM1. For 51 patients, both pre- and postoperative images were available, and thus, they were included in this study. Patients were divided into three age groups: children [under 10 years of age (*n*=19)], adolescents [10–19 years old (*n*=13)], and adults [over 19 years of age (*n*=19)]. The mean age of the children was 5.1 ± 2.0 years and that of the adolescents was 14.2 ± 2.5 years. The mean age of the adults was 41.5 ± 14.1 years. The cohort of the children comprised 12 boys and 7 girls. The cohort of the adolescents consisted of 3 male and 10 female patients and that of the adults consisted of 3 male and 16 female patients. A standard foramen magnum decompression (FMD) with resection of the C1 lamina and with either dissection of the dural outer layer (in 39 cases) or with dural opening and duraplasty (in 12 cases) was performed for everyone. The technique was chosen according to the surgeons’ preferences. The cerebellar tonsils were left intact in all cases. Five patients whose subjective symptoms did not improve or worsened after the first surgery required reoperation. The postoperative results were evaluated after the second surgery in these patients. Thus, postoperative evaluation in all the patients was performed after their Chiari treatment was considered complete, and no new operations were planned. The mean follow-up time was 7 years and 1 month (range 18–179 months). At the last evaluation of the medical records, no patient needed further surgical treatment, although some patients had further follow-up visits scheduled.

### Magnetic resonance imaging

The MRI scans were performed on either 1.5 or 3 T MRI scanners. The preoperative scans were made in Oulu University Hospital or the referring central hospitals, and the postoperative control scans were made in Oulu University Hospital. Standard sagittal T1-weighted sequences of the brain and both sagittal and axial T2-weighted sequences of the cervical spine were included in all the patient scans. The preoperative and postoperative brain or cervical MRIs were retrospectively analyzed. For the postoperative measurements, the last available control brain or cervical MRI was used. The median time interval between the surgery and the index postoperative imaging was 29 months (range 3–108 months) in children, 37 months (range 7–78 months) in adolescents, and 14 months (range 1–142 months) in adults. In 31 patients, only one postoperative MRI was performed.

### Radiological measurements

**Fig. 1:**
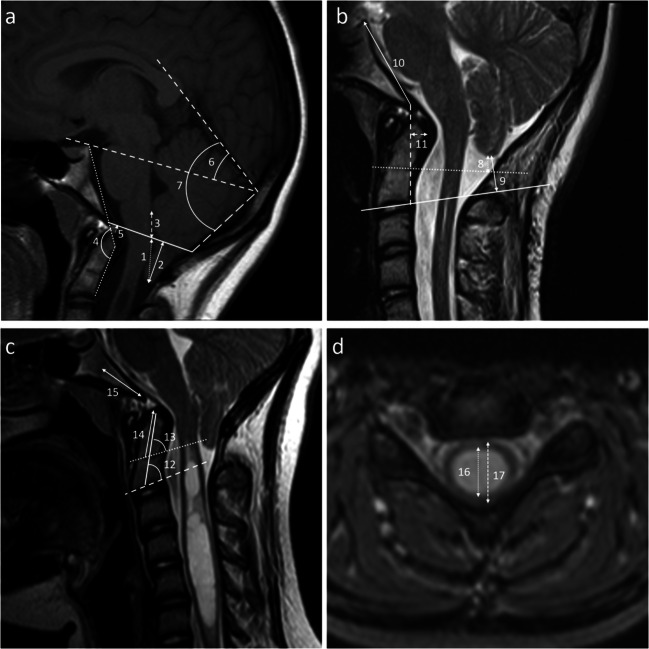
Representative images of the measurements on the T1-weighted (T1W) midsagittal (**a**), T2-weighted (T2W) midsagittal (**b** and **c**), and T2W axial (**d**) planes. **a** 1 = vertical distance of the tonsils to the midpoint of the McRae line; 2 = perpendicular distance of the tonsils below the McRae line; 3 = distance from the obex to the midpoint of the McRae line; 4 = angle between the line extending from the top of the dorsum sellae to the basion and the line between the inferodorsal portion of C2 to the most superodorsal part of the dens; 5 = vertical distance of the tip of the dens to the McRae line; 6 = angle between the tentorium cerebelli and a line from the internal occipital protuberance to the tuberculum sellae (Twining’s line); 7 = angle between the tentorium cerebelli and a line from the internal occipital protuberance to the opisthion (slope of the tentorium cerebelli). **b** 8 = perpendicular distance of the tonsils to the line drawn from the synchondrosis of the C2 vertebra; 9 = perpendicular distance of the tonsils to the line drawn from the end plate of the C2 vertebra; 10 = clivus length (distance from the basion to the posterior clinoid process); 11 = perpendicular distance of the most posterior extent of the odontoid process at the dural interface to a line drawn between the basion and the posterior aspect of the C2 vertebral body (pB-C2 line). **c** 12 = the angle between the line drawn from the end plate of the C2 vertebra and its intersection with a line drawn from the odontoid tip; 13 = the angle between the line drawn from the synchondrosis of the C2 vertebra and its intersection with a line drawn from the odontoid tip; 14 = length of the odontoid process; 15 = basiocciput length (distance from the basion to the sphenooccipital synchondrosis). **d** 16 = anteroposterior diameter of the syrinx; 17 = anteroposterior diameter of the spinal cord at the level of the syrinx

Seventeen different parameters were measured from the preoperative MRI and 11 from the postoperative MRI. The measured parameters are listed in Table 1, and the measurements are illustrated in Fig. 1. The diameter of the spinal cord was measured at the level of the syrinx’s widest area if the patient had syringomyelia. The measurements were planned by a pediatric radiologist (MS-P, with 16 years’ radiology experience), according to the radiological measurement methodology described by Tubbs et al. [37]. The measurements were conducted by two of the authors (M-PJ, radiology resident and JuN, radiologist), who were blinded to each other’s measurements. The mean values of the MRI parameters were calculated from the measurements made by two of the authors (M-PJ and JuN), and the interrater reliability was evaluated. Two methods were used to measure the cerebellar tonsils’ descent below the McRae line in the preoperative imaging: the distance vertical to the McRae middle point (Fig. 1a, measurement 1) and the distance perpendicular to the McRae line (Fig. 1a, measurement 2). Postoperative tonsillar herniation could not be evaluated from the McRae line because the occipital bone had been removed from the skull base. Thus, tonsillar descent was evaluated by measuring the distance from the tonsillar tip to the line drawn through the synchondrosis of the second cervical vertebra (C2) (Fig. 1b, measurement 8) and parallel to the lower end plate of the C2 vertebra on the pre- and postoperative images to assess the dynamics of the tonsillar tip location (Fig. 1b, measurement 9). Altogether, the tonsillar tip location was measured using four different measurement methods on the preoperative MRI images and two measurement methods on the postoperative MRI images.

**Table 1:** Preoperative MRI measurements in adults, adolescents, and children

	Adults Mean (CI)	Adolescents Mean (CI)	Children Mean (CI)
Cerebellar tonsils location in relation to:			
•1 = McRae line (vertical to middle point), mm	18.3 (15.9–20.8)	21.2 (16.7–25.6)	18.1 (15.2–21.0)
•2 = McRae line (perpendicular), mm	15.1 (12.1–18.2)	20.0 (15.2–24.7)	16.8 (13.6–19.9)
•8 = C2 synchondrosis, mm	3.3 (0.9–5.8)	1.6 (−2.9 to 6.0)	−1.7 (−5.0 to 1.5)
•9 = C2 vertebral end plate, mm	12.5 (10.1–14.9)	8.8 (4.9–12.7)	5.4 (2.3–8.4)
3 = McRae line – obex, mm	15.5 (13.9–17.2)	15.8 (12.3–19.3)	16.6 (14.8–18.5)
4 = Clivus canal angle, degrees	153.2 (148.6–157.9)	150.5 (140.2–160.8)	156.0 (151.0–161.0)
5 = McRae line – tip of the dens, mm	4.9 (3.7–6.1)	4.8 (3.7–5.9)	6.0 (5.0–7.1)
6 = Tentorium angle, mm	41.7 (39.8–43.6)	40.0 (37.7–42.3)	41.1 (39.5–42.8)
7 = Occipital angle, degrees	93.8 (91.1–96.4)	91.2 (86.6–95.7)	86.1 (83.3–88.8)
10 = Clivus length, mm	39.6 (37.2–42.1)	37.3 (34.9–39.6)	34.0 (32.5–35.5)
11 = pB-C2 line, mm	6.3 (4.8–7.8)	6.3 (4.9–7.6)	3.9 (3.0–4.8)
12 = Angle of the dens and the C2 vertebral end plate, degrees	68.0 (65.6–70.4)	65.8 (62.1–69.5)	72.7 (69.2–76.1)
13 = Angle of the dens and the C2 synchondrosis, degrees	77.3 (74.2–80.3)	73.7 (70.1–77.4)	76.9 (74.3–79.6)
14 = Odontoid process length, mm	18.7 (17.4–20.0)	19.3 (18.1–20.4)	15.5 (14.2–16.9)
15 = Basioccipital length, mm	20.2 (18.0–22.3)	22.1 (20.1–24.1)	17.6 (16.7–18.6)
16 = AP diameter of syrinx, mm	7.4 (5.6–9.8)	7.0 (4.3–9.8)	7.3 (3.2–11.3)
17 = AP diameter of spinal cord, mm	10.2 (8.9–11.6)	10.7 (8.5–12.9)	9.3 (6.4–12.2)

*AP* anteroposterior, *CI* confidence interval, *pB-C2 line* perpendicular distance to the basion-C2 vertebral line

The number in front of each classification refers to the measurement method shown in Fig. 1

### Clinical symptoms and findings

Data on symptoms and other clinical information were gathered from the Oulu University Hospital patient medical records. A neurosurgeon (NS, 16 years’ neurosurgery experience) evaluated from the patient records what symptoms or findings had been the indications for the surgery. The surgical results were assessed as a change in these symptoms. According to the patients’ medical records, the symptoms that were considered to be related to CM1 and thus were mentioned as indications for surgery were headache, neck pain, paresthesia, pain in limbs, limb weakness, balance problems, various visual disturbances, vertigo, and nausea. Syringomyelia and, in children, scoliosis appeared to be an indication for surgery in all cases if present. Preoperative motor and somatosensory evoked potentials, which were evaluated in 13 children, three adolescents, and only one adult patient, were not included in this study.

### Statistical analysis

According to the interrater reliability analysis, there was moderate to excellent reliability in all but one measurement value between the two measurers. Poor reliability between the two measurers was only observed in analyzing the basilar invagination measurement (interclass correlation coefficient [ICC] 0.485). In the analysis of the other measurements, the ICC varied between 0.510 and 0.970. An ANOVA test was used to evaluate the differences in the pre- and postoperative measurements between the surgical outcome groups. A chi-square test was used to evaluate whether preoperative categorical variables were associated with postoperative symptom improvement. A repeated measures ANOVA was used to compare pre- and postoperative measurements. Children, adolescents, and adults were analyzed separately except when clinical symptoms were studied due to relatively small number of cases per outcome group. A cerebellar tonsillar movement upward or downward was reported if the difference was more than 2 mm. For all the tests, a significance level of < 0.05 was used. All data were analyzed using IBM SPSS Statistics 25.0 software (IBM Corp., Armonk, NY, USA).

## Results

According to their medical records, three of 19 (15.8%) children and four of 13 (30.8%) adolescents were asymptomatic but had either syringomyelia or/and scoliosis, which were considered to be indications for surgery. Three children had syringomyelia, and two had both syringomyelia and scoliosis. In adolescents, two had scoliosis, seven had syringomyelia, and two had both. There were no asymptomatic adult patients. Syringomyelia was found in 42.1% (*n* = 8) of the adults, in 69.2% (*n* = 9) of the adolescents, and in 26.3% (*n* = 5) of the children. The preoperative syrinx diameter varied from 3.1 to 12.3 mm in children, from 1.5 to 13.8 mm in adolescents, and from 3.8 to 9.8 mm in adults. Seventeen out of 22 (77.3%) patients with syringomyelia were female. None of the adults had scoliosis, while two children (10.5%) and four adolescents (30.8%) had scoliosis. Only in one patient was scoliosis an incidental finding that did not influence decision-making. Scoliosis (scoliometer > 6 degrees or Cobb angle > 20 degrees) appeared to be an indication for CM1 surgery in five other cases. Syringomyelia was always considered an indication to perform surgery.

### Preoperative radiological evaluation

The MRI measurements of the children, adolescents, and adults were evaluated separately (Table 1). In the children, the cerebellar tonsils were located more caudally when measured from the C2 vertebral end plate compared to adults. The clivus length, the basioccipital length, the odontoid process length, the pB-C2 line, and the occipital angle were smaller in children. However, the angle of the dens and the C2 vertebral end plate was bigger in children.

There was no statistically significant difference between the two different tonsillar location measurement methods taken from the McRae line (Fig. 1a, measurements 1 and 2). The tonsillar tip location perpendicular to the McRae line was on average 16.8 mm (range 7.7–30.9 mm) in the children, 20.0 mm (range 10.6–41.7 mm) in the adolescents, and 15.1 mm (range 5.3–30.0 mm) in the adults. The tonsillar tip perpendicular location measured from the McRae line correlated well to the location measured from the C2 vertebra using both the synchondrosis and the end plate line (*r* = 0.771 and *r* = 0.741, respectively, *p* < 0.001).

### Postoperative radiological evaluation

In the children, the postoperative measurements of the following parameters showed a statistically significant increase compared to the preoperative measurements: the angle of the dens and the C2 end plate, the basioccipital length, the clivus length, the odontoid process length, and the pB-C2 line. In the adults, both the tonsillar location to the C2 end plate line and the tonsillar location to the C2 synchondrosis line increased. While the same tendency was observed in the children, it was not statistically significant. In the children, the adolescents, and the adults, the diameters of the syrinx decreased after the operation. Furthermore, the diameter of spinal cord decreased after the operation in both the adolescents and the adults. In the children, the diameter of spinal cord had a tendency of decreasing after the operation, but a statistically significant change was not observed. These measurement results are shown in detail in Table 2.

**Table 2:** The measurements that changed significantly after surgery when comparing preoperative and postoperative MRI

	Children		Adolescents		Adults	
	Mean difference (CI)	*p*-value	Mean difference (CI)	*p*-value	Mean difference (CI)	*p*-value
Cerebellar tonsils location, mm						
•C2 vertebral end plate	4.4 (−0.3, 9.2)	0.066	1.0 (−3.0, 5.0)	0.597	3.8 (1.7, 6.0)	0.002
•C2 synchondrosis	4.1 (−0.1, 8.3)	0.054	0.1 (−4.5, 4.7)	0.967	5.4 (2.3, 8.5)	0.002
Angle of the dens and the C2 vertebral end plate, degrees	−4.5 (−8.7, −0.2)	0.040	−2.8 (−6.0, −0.3)	0.071	0.7 (−1.6, 3.0)	0.538
Basioccipital length, mm	3.7 (2.3, 5.1)	< 0.001	1.0 (−0.4, 2.5)	0.145	0.6 (−0.9, 2.2)	0.406
Clivus length, mm	4.3 (2.6, 6.0)	< 0.001	1.0 (−1.3, 3.2)	0.333	0.3 (−1.3, 2.0)	0.699
Odontoid process length, mm	2.9 (1.6, 4.2)	< 0.001	0.5 (0.0, 1.1)	0.048	0.8 (−0.4, 1.9)	0.165
pB-C2 line, mm	2.3 (1.3, 3.2)	< 0.001	0.2 (−0.2, 0.6)	0.237	0.1 (−0.9, 1.1)	0.823
Diameter of the spinal cord, mm	−1.8 (−4.1, 0.4)	0.091	−3.4 (−5.6, −1.2)	0.008	−2.5 (−3.7, −1.4)	0.001
Diameter of the syrinx, mm	−3.8 (−6.0, −1.6)	0.009	−3.6 (−6.1, −1.0)	0.013	−4.0 (−6.5, −1.6)	0.006

*CI* confidence interval, *pB-C2 line* perpendicular distance to the basion-C2 vertebral line

After the operation, the tonsils were located more caudally when measured perpendicular to the C2 end plate line in the adolescents with syringomyelia, 7.3 mm (2.9–11.7 mm), compared to those without syringomyelia, 15.3 mm (5.8–24.9 mm), *p* = 0.042. The opposite finding was observed in the adults where tonsils were located more caudally without syringomyelia, 6.2 mm (3.7–8.7 mm) vs in the adults with syringomyelia 11.7 mm (6.3–17.0 mm) (*p* = 0.026) when measured perpendicular to the C2 synchondrosis line. The angle of the dens and the C2 synchondrosis was smaller in the adults without syringomyelia, 74.5 degrees (71.7–77.2 degrees), compared to those with syringomyelia, 80.1 mm (73.9–86.2 mm), *p* = 0.043. In the adolescents, the clivus canal angle was smaller on the preoperative imaging (*p* = 0.022) in the presence of syringomyelia. The same tendency was noticeable (*p* = 0.081) in these patients postoperatively. No other significant differences were observed in the pre- or postoperative measurements between the patients with or without syrinxes. The preoperative size of the syrinx did not correlate with the change in the size of the syrinx at the first or the second follow-up imaging after surgery. The time interval between the surgery and the postoperative imaging did not correlate with the change in the size of the syrinx or the change in the position of the cerebellar tonsils relative to the C2 vertebral end plate or the C2 synchondrosis. In 90.9% (*n* = 20) of the patients with syringomyelia, the size of the syrinx was smaller postoperatively than preoperatively. The syrinxes increased in size after the operation in two out of 22 patients (9.1%) with syringomyelia. In one patient, the syrinx had increased at the first control scan and had decreased at the second control scan, remaining larger compared to the preoperative imaging (follow-up time 72 months). In this patient, there was a 13 months’ time lapse between the last preoperative imaging and the surgery, leaving the possibility that the syrinx had already increased in size preoperatively. In the other patient, the syrinx had decreased at the first postoperative imaging and increased at the second follow-up imaging 11 months after the surgery.

**Fig. 2:**
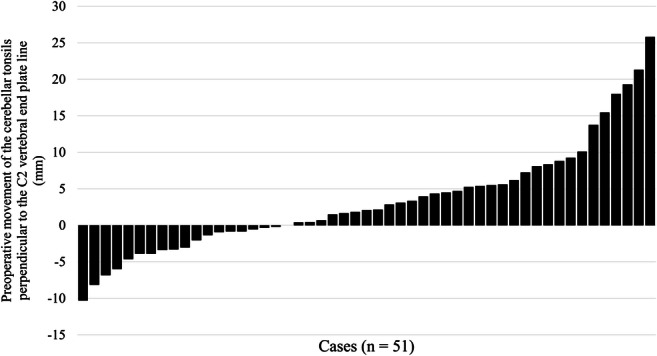
Movement of the cerebellar tonsils in relation to the C2 vertebral end plate after surgery. A positive value means that the tonsils have moved upward

Figure 2 shows the changes in the positions of the cerebellar tonsils relative to the C2 vertebral end plates after surgery. In most cases, the tonsils were located more cranially after surgery. The margin of error was considered to be 2 mm for a change of the cerebellar tonsils’ location. The tonsils moved cranially postoperatively in 51.0% (*n* = 26), did not change in 27.4% (*n* = 14), and moved caudally in 21.6% (*n* = 11) of the patients.

### Postoperative behavior of clinical symptoms, scoliosis, and syringomyelia

In 46 patients, only one operation was performed, and in five patients (two children, two adolescents, and one adult), reoperation was performed. No patient required more than two operations. The postoperative results were evaluated after the treatment was considered complete, and no more operations were planned.

The surgical outcomes were evaluated, and the patients were divided into four subgroups according to the postoperative behavior of the preoperative clinical symptoms: resolved, improved, remained unchanged, or worsened. In the patients with scoliosis or syringomyelia who did not have subjective symptoms, the results of the surgery were estimated according to the changes in the syringomyelia and/or scoliosis measurements after the operation. In most of the patients, the subjective clinical symptoms that were indications for the surgery improved or resolved (Tables 3 and 4). One initially subjectively asymptomatic child, with scoliosis and severe syringomyelia being indications for surgery, had worsened scoliosis, and her syringomyelia did not improve after the surgery, which was performed when the patient was 14 years old (Table 3). This patient underwent surgery for shunting of the syrinx 1.5 years later. During the following 10 years, she progressively developed symptoms related to syringomyelia in spite of the decrease of the syrinx. None of the patients with scoliosis required surgery due to it; however, four out of six patients were treated with a scoliosis brace.

**Table 3:** Surgical outcomes in children, adolescents, and adults

Symptoms and/or Scoliosis	Children *n* (%)		Adolescents *n* (%)		Adults *n* (%)	
	With syrinx	Without syrinx	With syrinx	Without syrinx	With syrinx	Without syrinx
Resolved	1 (20.0)	6 (42.9)	2 (22.2)	1 (25.0)	1 (12.5)	2 (18.2)
Improved	4 (80.0)	7 (50.0)	5 (55.6)	3 (75.0)	6 (75.0)	7 (63.6)
Remained unchanged	0	1 (7.1)	1 (11.1)	0	1 (12.5)	2 (18.2)
Worsened	0	0	1 (11.1)	0	0	0

**Table 4:** Surgical outcomes in patients with subjective symptoms

Symptoms	Children *n* (%)	Adolescents *n* (%)	Adults *n* (%)	Total *n* (%)
Resolved	7 (43.8)	2 (22.2)	3 (15.8)	12 (27.3)
Improved	8 (50.0)	6 (66.7)	13 (68.4)	27 (61.4)
Remained unchanged	1 (6.3)	1 (11.1)	3 (15.8)	5 (11.4)
Worsened	0	0	0	0

Due to the small sizes of the four subgroups concerning the behavior of the subjective clinical symptoms, scoliosis, or syringomyelia, the patients were divided into two outcome groups for statistical analysis: symptoms improved/resolved and symptoms remained unchanged/worsened (Table 4). Table 5 shows the clinical symptoms that were considered indications for surgery.

**Table 5:** Subjective clinical symptoms (grouped for simplification) considered indications for surgery according to medical records

	Children *n* (%)		Adolescents *n* (%)		Adults *n* (%)		Total *n* (%)	
	Present preoperatively	Improved/resolved	Present preoperatively	Improved/resolved	Present preoperatively	Improved/resolved	Present preoperatively	Improved/resolved
Headache	14 (73.7)	13 (92.9)	9 (69.2)	6 (66.6)	15 (78.9)	13 (86.7)	38 (74.5)	32 (84.2)
Motor or sensory disturbances in the limbs	3 (15.8)	3 (100.0)	3 (23.1)	3 (100.0)	12 (63.2)	10 (83,3)	18 (35.3)	16 (88.9)
Balance disturbances	1 (5.3)	1 (100.0)	2 (15.4)	2 (100.0)	5 (26.3)	4 (80.0)	8 (15.7)	7 (87.5)
Visual disturbances	2 (10.5)	2 (100.0)	1 (7.7)	1 (100.0)	0	N/A	3 (9.7)	3 (100.0)
Other symptoms^a^	1 (3.2)	1 (100.0)	0	N/A	1 (5.0)	1 (100.0)	2 (3.9)	2 (100.0)

*N/A* not applicable

^a^Other symptoms were fatigue in one adult patient and vomiting in one child

The preoperative diameter of the spinal cord at the syrinx level was smaller in the patients whose symptoms resolved or improved (mean difference 3.3 mm, 95% CI: 0.5–6.2 mm, *p* = 0.024). However, the preoperative size of the syrinx itself (Table 1, measurement 16) did not differ between the two surgical outcome groups. The preoperative presence of a syrinx or scoliosis was not associated with postoperative symptom improvement (*p* = 1.00). The postoperative movement of the cerebellar tonsils (perpendicular to the C2 end plate or the C2 synchondrosis) compared to the preoperative imaging was not associated with symptom improvement (*p* = 0.468 and *p* = 0.372, respectively). Similarly, the change in the size of the syrinx after surgery was not associated with symptom improvement, *p* = 0.363. There were no statistically significant differences between the two surgical outcome groups in any other measurement results.

All the children and all but one adolescent were operated with FMD without duraplasty, while in adults, duraplasty was done in over half of the operations. Thus, the effect of the operative method on surgical outcome in different age groups could not be statistically analyzed.

## Discussion

In the present study, we could not define any single parameter measured on the preoperative MRI that would reliably predict change in the clinical symptoms after FMD surgery. This is in line with the previous studies [4, 11, 14].

The cerebellar tonsils are reported to move during neck extension and flexion and during the cardiac cycle [9, 35]. Thus, there could be a margin of error, which should be considered especially in borderline cases when tonsils are located only 5 to 6 mm below the foramen magnum. In the present study, in only three cases were the cerebellar tonsils located just 5–8 mm below the McRae line preoperatively. In all the other cases, the tonsils were located more caudally. All four methods used in this study to measure the cerebellar tonsils’ descent were feasible and correlated well with each other. Thus, the postoperative change in the tonsillar location can be evaluated as the distance from the C2 vertebral end plate or the C2 synchondrosis.

Although the preoperative imaging parameters did not show a statistically significant correlation with the improvement of symptoms, the cerebellar tonsils moved more cranially after FMD surgery in half of the patients, which was a positive radiological result of the surgery. This is an especially interesting finding, since tonsillar coagulation was performed only for one patient. Previously, it has been reported that in most cases, the cerebellar tonsils moved upward or backward after posterior fossa decompression [40]; in the same study, the maximal syrinx/spinal cord ratio and the syrinx length were demonstrated to improve after surgery. In addition, the postoperative tonsillar movement cranially was shown to correlate with the syrinx/spinal cord ratio [40]. We did not find a statistically significant correlation between symptom improvement and changes in syrinx size or tonsillar movement after surgery. The change in the size of the syrinx did not correlate to the tonsillar movement postoperatively. In three asymptomatic children and four asymptomatic adolescents, syringomyelia with or without scoliosis was considered to be a sufficient finding to make a decision for an operation, which was in accordance with the standard practice [41]. Neither changes in syrinx size nor changes in tonsillar location correlated with the time lapse between the surgery and the postoperative MRI, despite the big differences in follow-up times between the patients.

In the children, the clivus canal angle, the angle of the dens and the C2 end plate, the basioccipital length, the clivus length, the odontoid process length, the pB-C2 line, and the syrinx significantly changed postoperatively. However, the changes in some of these parameters may be explained by the child’s head growth, since the time between the preoperative and postoperative imaging was on average 48 months (range 8 months–9 years and 5 months). Skull growth could explain the difference between some linear measurement results when comparing preoperative MRI to postoperative imaging in children. In the adults, the tonsillar location in relation to the C2 end plate line and the C2 synchondrosis line, as well as the diameters of the spinal cord and the syrinx, changed after surgery. In the adolescents, only the odontoid process length and the diameters of the spinal cord and the syrinx changed after surgery.

In our study, 42.1% of the adults, 69.2% of the adolescents, and 26.3% of the children had preoperative syringomyelia. The prevalence of syringomyelia was in accordance with previous studies, which showed the presence of syringomyelia in 20–70% of cases [8, 18, 33, 36]. Interestingly, syringomyelia predominated in female patients, being present in 52% of females and only 28% of males. Most of the syrinxes decreased postoperatively in all age groups of the patients. Only one patient in this population required syrinx shunting. Although Hekman et al. found a correlation between the presence of a syrinx and postoperative symptom improvement [15], we did not find this correlation, which is in concordance with the studies by Atchley et al. and Kalb et al. [4, 18]. Correlation of a decrease in the size of the spinal cord diameter with symptom improvement after surgery has been shown previously [12]. In our study, there was a tendency for symptom improvement in patients whose spinal cord size at the level of the syrinx was smaller preoperatively. However, due to the few patients included (only three patients with syringomyelia whose symptoms did not improve in our study), conclusions cannot be drawn with certainty.

The prevalence of scoliosis is reported to vary between 13 and 36% in patients with CM1 [8, 30, 34]. In the present study, 10.5% of the children and 30.8% of the adolescents had scoliosis, while scoliosis was not mentioned in any medical records of the adults. The prevalence of scoliosis has been shown to be higher in CM1 patients with syringomyelia [30]. However, in our study, we did not find this correlation, probably due to small number of patients. We also found no difference in postoperative symptom improvement between patients with or without scoliosis. In a previous study, scoliosis with a Cobb angle < 20 degrees was associated with better postoperative stability/improvement [20].

Ladner et al. reported that a pB-C2 line length over 3 mm, reflecting ventral canal encroachment on preoperative MRI, was associated with postoperative symptom improvement [21]. In contrast, Greenberg et al. found the pB-C2 line length to be insignificant [14]. In our study, this measurement did not correlate with symptom improvement (*p* = 0.593), and it increased postoperatively only in children. It has been reported that after posterior fossa decompression, the pB-C2 line increases probably due to lower pressure on the ventral dura [5]. Like previous studies, we did not find a statistically significant association with other imaging parameters concerning the clivus canal angle, the dens angle, the tonsillar location, the obex position, the tentorial angle, and the presence of a syrinx or scoliosis [4, 11, 14].

The postoperative clinical results were considered good in almost all the cases. In 12% of the patients, there was no improvement in the subjective symptoms. In other studies, approximately 30–40% of patients’ symptoms did not improve, or the improvement was quite small [2, 12, 14, 18, 25, 27]. Patients may also experience symptom recurrence after a long follow-up period [26]. We cannot directly compare our surgical outcome to previous studies, as we only evaluated the changes in the symptoms that were considered to be an indication for surgery. Patients might also have had other symptoms that were relieved after surgery, but preoperatively they were either not mentioned in the medical records or they were not considered to be related to CM1.

The decision for CM1 surgery should be based on clinical evaluation rather than imaging findings only. Mild symptomatic CM1 may improve spontaneously in both adults and children [7, 19], although greater spontaneous symptom improvement is more likely to happen in children [19]. Asymptomatic CM1 often remains asymptomatic [6, 22, 23, 39]. In a study in which CM1 patients were followed radiologically without any surgery, no significant worsening in asymptomatic patients without syringomyelia was found [39]. According to another study, symptom development did not seem to correlate with radiological changes, and routine imaging follow-up in asymptomatic cases without syringomyelia was not considered reasonable [39]. The potential benefits of surgery and MRI follow-up must always outweigh the potential disadvantages. Because the postoperative symptom improvement of CM1 patients did not correlate with any imaging parameter in our study, we do not recommend routine follow-up imaging in cases with symptom improvement. Although syringomyelia can often remain asymptomatic [29], it can also be associated with severe symptoms, and previously asymptomatic patients may have a sudden onset of symptoms [32]. Therefore, we recommend follow-up imaging in CM1 patients with syringomyelia.

Our study has a few limitations. The sample size was quite small when the patients were divided into four groups according to surgical outcome. Therefore, the patients were divided into two different outcome groups for the final statistical analyses. A small sample size can lead to bias, and the significance of chance may be emphasized. Poor reliability between the two measurers was observed in the measurement of the tip of the dens location, which can be considered a limitation for making conclusions about the significance of this particular finding. The patients’ surgical outcomes were retrospectively evaluated based on their medical records, which may cause inaccuracy. Due to the retrospective nature of this study and the relatively small sample size, the clinical symptoms could not be scored. In addition, we did not undertake any multiparametric analysis of the symptoms. Thus, some MRI parameters or combinations of parameters could still be predictive of the easing of symptoms. Further prospective studies are needed to answer these questions.

At our institution, until the last few years neither cerebrospinal fluid (CSF) flow imaging by cine MRI nor intraoperative ultrasound was used routinely in Chiari patients. There was only a visual, subjective evaluation of the CSF flow restoration at the level of foramen magnum during the surgery. In the patients without duraplasty, the dural dissection always resulted in such thinning of dura that the movement of cerebellar tonsils and the restoration of CSF flow were visually confirmed. Nowadays, the CSF cine sequence is included in the MRI protocol, and intraoperative ultrasound is used whenever necessary.

## Conclusions

In half of the operated CM1 patients, the cerebellar tonsils shifted upward, with the distance to the C2 synchondrosis or the C2 end plate being equally feasible for evaluation of these changes. Also, the syrinxes decreased in size postoperatively in most patients. However, we found no reliable MRI parameters that could predict surgical outcome in CM1 patients from the preoperative imaging nor parameters that could reflect the surgical outcome from the postoperative imaging. Patient selection for CM1 surgery should be considered thoroughly based on clinical symptoms rather than any single radiological parameter after CM1 diagnosis established on MRI. Postoperative MRI imaging after Chiari operations has no benefit in cases without syringomyelia or suspected complications.
